# Comparison of paraspinal muscle composition measurements using IDEAL fat–water and T2-weighted MR images

**DOI:** 10.1186/s12880-023-00992-w

**Published:** 2023-03-30

**Authors:** Sara Masi, Meaghan Rye, Alexa Roussac, Neda Naghdi, Brent Rosenstein, Jeannie F. Bailey, Maryse Fortin

**Affiliations:** 1 Department of Health Kinesiology and Applied Physiology, Concordia University, 7141 Sherbrooke Street W, SP-165.29, Montreal, QC H4B 1R6 Canada; 2 Department of Orthopaedic Surgery, University of California, San Francisco, CA USA; 3 PERFORM Centre, Concordia University, Montreal, QC Canada; 4 Centre de Recherche Interdisciplinaire en Réadaptation (CRIR), Montreal, QC Canada

**Keywords:** Fatty infiltration, IDEAL, Multifidus, Erector spinae, Psoas major, Paraspinal muscle, Magnetic resonance imaging

## Abstract

**Purpose:**

The purpose of this study was to evaluate the agreement between paraspinal muscle composition measurements obtained from fat–water images using % fat-signal fraction (%FSF) in comparison to those obtained from T2-weighted magnetic resonance images (MRI) using a thresholding method.

**Methods:**

A sample of 35 subjects (19 females, 16 males; 40.26 ± 11.3 years old) was selected from a cohort of patients with chronic low back pain (LBP). Axial T2-weighted and IDEAL (Lava-Flex, 2 echo sequence) fat and water MR images were obtained using a 3.0 Tesla GE scanner. Multifidus, erector spinae, and psoas major muscle composition measurements were acquired bilaterally at L4–L5 and L5–S1 using both imaging sequences and related measurement methods. All measurements were obtained by the same rater, with a minimum of 7 days between each method. Intra-class correlation coefficients (ICCs) were calculated to assess intra-rater reliability. Pearson Correlation and Bland–Altman 95% limits of agreement were used to assess the agreement between both measurement methods.

**Results:**

The intra-rater reliability was excellent for all measurements with ICCs varying between 0.851 and 0.997. Strong positive correlations indicating a strong relationship between composition measurements were obtained from fat–water and T2-weighted images for bilateral multifidus and erector spinae muscles at both spinal levels and the right psoas major muscle at L4–L5, with correlation coefficient *r* ranging between 0.67 and 0.92. Bland–Altman plots for bilateral multifidus and erector spinae muscles at both levels revealed excellent agreement between the two methods, however, systematic differences between both methods were evident for psoas major fat measurements.

**Conclusion:**

Our findings suggest that utilizing fat–water and T2-weighted MR images are comparable for quantifying multifidus and erector spinae muscle composition but not of the psoas major. While this suggests that both methods could be used interchangeably for the multifidus and erector spinae, further evaluation is required to expand and confirm our findings to other spinal levels.

## Introduction

The paraspinal muscles play an important role in trunk stability providing dynamic support to the vertebral column [1, 2]. Substantial evidence revealed the presence of paraspinal muscle structure changes (e.g., atrophy, increased fatty infiltration) and functional deficits (e.g., reduced muscular strength and endurance) in subjects with chronic low back pain (LBP) [3, 4]. Of the paraspinal muscles, the lumbar multifidus (MF) and erector spinae (ES) muscles are the most commonly affected [5, 6]. Although less frequently examined, additional key spinal stabilizers such as the psoas major (PM) and quadratus lumborum (QL), may also contribute to LBP [2, 4, 6].

The presence of fatty infiltration in the paraspinal muscles is associated with an increased risk of developing persistent or recurrent LBP [7–9]. Moreover, fatty infiltration is linked to spinal pain and dysfunction, including decreased isometric muscle strength and postural control [3, 8, 9]. Therefore, there is a growing interest to quantify paraspinal muscle quality (e.g., composition) in order to better understand the etiology of LBP and the impact of different rehabilitation and therapeutic interventions in this patient population.

Magnetic resonance imaging (MRI) is the gold standard for assessing the morphology and composition (e.g., fatty infiltration) of paraspinal muscle due to its high imaging resolution and detailed soft tissue contrast, thereby allowing precise differentiation of muscle, fat, and bone structures [10, 11]. Quantitative and qualitative methods using MRI have been performed to assess the composition (e.g., fatty infiltration) of paraspinal muscles, including the MF and ES [11, 12]. The Goutallier Classification is a grading scale designed to qualitatively assess the amount of fat present within muscles [12]. Although qualitative assessment tools such as the Goutallier Classification are relatively simple and time efficient, their lack of measurement accuracy may limit their reproducibility [11–13]. In contrast, quantitative measures of paraspinal fat infiltration can be obtained from chemical shift fat and water images (e.g., DIXON, IDEAL), which offers superior accuracy to delineate muscle and fat tissues and is the current contemporary standard for evaluating skeletal muscle composition [1, 11, 14, 15]. Fat signal fraction (FSF) is based on the frequency emitted by fat and water protons within the region of interest (ROI) traced around each muscle and is calculated as follows: %FSF = (Signal_fat_/[Signal_water_ + Signal_Fat_] × 100) [1, 10, 11]. Alternatively, quantitative paraspinal muscle composition measurements can also be obtained from T1-weighted and T2-weighted images using different thresholding techniques and fat measurement definitions [11, 14]. For example, functional cross-sectional area (FCSA, area of lean muscle mass), ratio of FCSA to total cross-sectional area (FCSA/CSA), fat cross-sectional area (fCSA), total CSA − FCSA, or signal intensity ratio measures have been used to assess muscle composition from T1- and T2-weighted MR images [11, 12, 16]. Both MR sequences provide accurate and reliable measurements of muscle composition and remain widely used in clinical and research settings due to their accessibility [9, 11, 15].

While chemical shift fat and water images, and T1- or T2-weighted images are both useful sequences for assessing paraspinal muscle composition [1, 11, 12, 14], the literature presents inconsistent findings regarding muscle composition measurements in relation to LBP. Variations in methodological approaches between imaging studies likely contribute to the inconsistent literature findings. Additionally, the definition of “fatty infiltration” varies across studies, making it difficult to replicate and compare findings [11]. As such, the agreement between data derived from both sequences remains to be established. Therefore, the purpose of the present study was to assess the agreement between paraspinal fatty infiltration measurements derived from IDEAL fat and water images using fat signal fraction in comparison to T2-weighted images using a thresholding technique. Paraspinal muscle composition measurements of the MF, ES and PM muscles were obtained with each method at the L4–L5 and L5–S1 spinal levels. We hypothesize that the agreement between the two methods will be excellent.

## Methods

### Study sample

This study included baseline MRI scans of 35 subjects (19 females, 39.95 ± 10.7 years old; 16 males, 40.63 ± 12.4 years old) selected from a larger patient cohort involved in a randomized controlled trial (NCT04257253, first registration date: 05/02/2020) evaluating the effect of two exercise therapy interventions on paraspinal muscle morphology and function. Inclusion criteria were: (1) non-specific chronic LBP (≥ 3 months) with or without leg pain, (2) had a “moderate” or “severe” score on the modified Oswestry Low Back Pain Questionnaire, (3) speak English or French, and (4) did not engage in sport or training specifically for the lower back musculature 3 months prior the beginning of the trial. Exclusion criteria included participants who were under 18 or over 65 years old, had signs of nerve root compression or motor deficits, had a history of spinal surgery or vertebral fractures, had significant structural abnormalities in the spine (such as spondylolisthesis or scoliosis greater than 10 degrees), were pregnant, or had comorbidities that could prevent them from safely participating in an exercise program. The project was approved by the Central Ethics Research Committee of the Quebec Minister of Health and Social Services (#CCER-19-20-09). Prior to any data collection, all subjects provided written informed consent in compliance with ethical standards. All methods were carried out in accordance with relevant guidelines and regulations.

### MRI protocol

Sagittal and axial T2-weighted (TR:3800, TE:98) and IDEAL (Lava-flex, 2 echo sequence, TE:4.5, TE: minimum full, flip angle:5) fat and water images of the entire lumbar spine (L1-L5) were obtained using a 3.0 Tesla GE scanner (Milwaukee, WI, USA) for a total acquisition time of about 7 min. A standard phased-array body coil with 16 channels was used, with 4-mm slice thickness, 180-mm^2^ field of view and 512 × 512 matrix.

### Muscle measurements

Bilateral MF, ES, and PM muscle composition measurements for every subject were obtained from axial images at mid-disc for L4–L5 and L5–S1. These two levels were selected as most paraspinal muscle morphological degenerative changes [13, 17] and spinal pathologies occur at the two lower lumbar levels [18]. Multi-planar reconstruction was used, if necessary, to correct the orientation of the MRI slice at mid-disc perpendicular to the muscle mass.

**Fig. 1:**
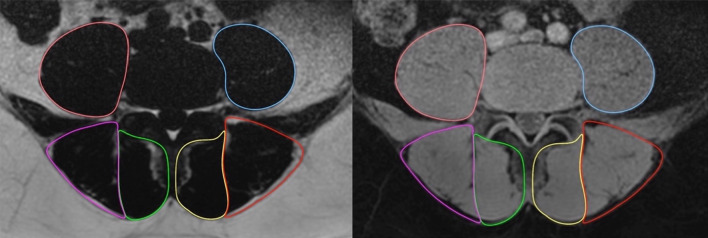
%FSF method. Example of ROI outlining the multifidus, erector spinae, and psoas major muscles using fat image (left) and water image (right)

Muscle composition measurements were first obtained using the water and fat axial images using the Horos DICOM viewer software (4.0.0). The ROI representing the CSA of a muscle of interest was traced manually around the individual muscles on the axial fat image and then copied onto the corresponding water image at each spinal level (Fig. 1). Related signal intensities were obtained from both fat and water images and used to calculate the individual muscle’s percentage fat signal fraction (%FSF) using the following formula: %FSF = (Signalfat/[Signalwater + SignalFat] × 100).

**Fig. 2:**
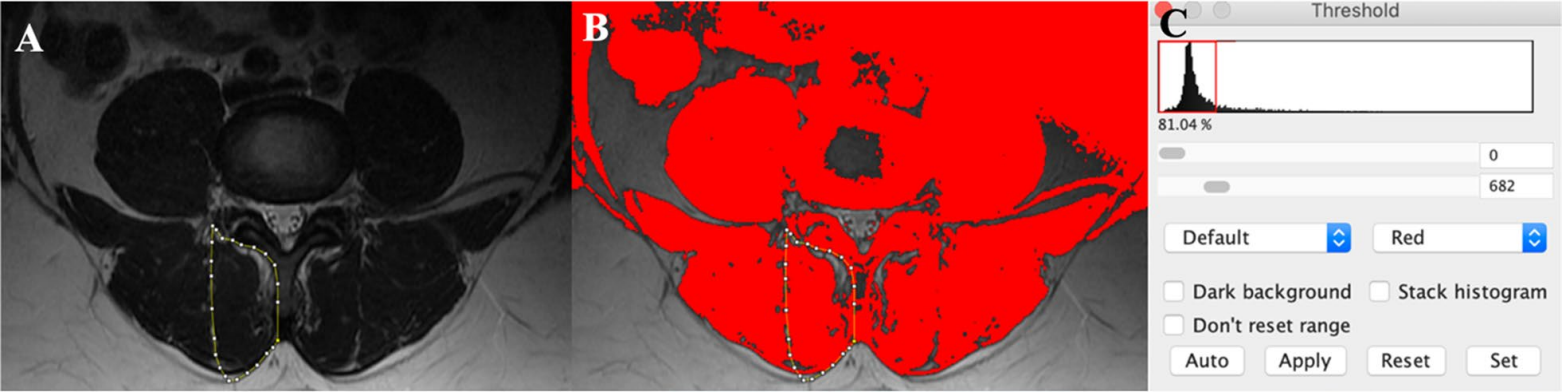
Thresholding method. **A** Outline of the total CSA of the right multifidus. **B** Outline of the multifidus demonstrating lean muscle cross-sectional area (FCSA) represented by the area highlighted in red. **C** Histogram function for selecting threshold value

Corresponding axial T2-weighted images were then used to obtain muscle composition measurements using a manual thresholding technique with ImageJ image analysis software (National Institutes of Health, Bethsda, Maryland) at the same spinal level. MF, ES, and PM muscle functional cross-sectional area (FCSA, area of lean muscle mass, excluding fatty infiltration) was measured by manually selecting a thresholding signal within the muscle total CSA using a histogram function to include only pixels of lean muscle (Fig. 2). The grayscale range representing the lean muscle mass was established for each subject and scan slice. This established thresholding technique is highly reliable [12, 16]. The related fat percentages of each muscle were calculated using the following formula: % fat = 1− [FCSA/CSA]). The PM measurements at L5–S1 were excluded in 5 patients due to poor image quality following correction of the orientation of the MRI slice.

### Statistical analysis

The mean and standard deviation of each muscle measurement at spinal levels L4–L5 and L5–S1 were computed as part of the descriptive statistics. The intra-rater reliability of fat measurements obtained using the %FSF and thresholding technique for all muscles was investigated using a sample of 10 random images. Intra-class correlation coefficient (ICC_3,1_) using a two-way random-effect model, single measure and absolute agreement was used. The ICCs and corresponding 95% confidence intervals were interpreted using the following guidelines, as suggested by Portney and Watkins: 0.00–0.49 = poor, 0.50–0.74 = moderate, and 0.75–1.0 = excellent [19]. The standard error of measurements (SEM) was also calculated to provide an estimate of the expected error related to each measurement method. Pearson’s correlation was used to evaluate the relationship between muscle composition measurements obtained with the %FSF and thresholding technique. According to Cohen's guidelines, the strength of the correlation coefficients (r) was interpreted in the following way: r = 0.10 as weak, r = 0.30 as moderate, and r = 0.50 as strong [20]. Agreement between both measurement techniques was also evaluated using the Bland and Altman method by calculating the mean difference and 95% limits of agreement. The statistical analysis was conducted using Statistical Package for the Social Sciences version 28.0 (SPSS Inc, Chicago, Illinois).

## Results

### Subjects

The demographic characteristics of the 35 subjects are presented in Table 1. The mean values and standard deviations (SD) of age, height, weight, body mass index and LBP duration was 40.26 ± 11.3 years, 171.29 ± 9.8, 76.57 ± 19.3 kg, 26.08 ± 5.4 kg/m^2^ and 88.50 ± 91.4 months respectively.

**Table 1:** Subjects’ demographic characteristics

	All (n = 35)	Female (n = 19)	Male (n = 16)
Age (year)	40.26 ± 11.3	39.95 ± 10.7	40.63 ± 12.4
Height (cm)	171.29 ± 9.8	166.00 ± 8.6	177.56 ± 7.2
Weight (kg)	76.57 ± 19.3	67.74 ± 10.7	87.06 ± 22.2
BMI (kg/m^2^)	26.08 ± 5.4	23.87 ± 5.2	27.52 ± 5.5
LBP duration (months)	88.50 ± 91.4	75.94 ± 73.0	102.63 ± 109.3

Values expressed as mean ± SD (range)

*BMI* body mass index, *LBP* low back pain

### Intra-rater reliability

In preparation for this study, the rater (SM) received training from an experienced rater (MF) to identify muscle borders and performed related segmentations. Intra-rater reliability of the rater (SM) was verified using a random sample of 10 fat–water images followed by corresponding T2-weighted MR images. The intra-rater reliability and SEM results are presented in Table 2. The ICCs ranged from 0.851 to 0.997 indicating excellent intra-rater reliability for all fat measurements obtained via both methods. In general, the SEM was greater for the thresholding measurements as compared with %FSF measurements.

**Table 2:** Intra-rater reliability and 95% confidence interval (CI) for %FSF and thresholding measurements

	%FSF measurements N = 10		Thresholding measurements N = 10	
	ICC [95% CI]	SEM (%)	ICC [95% CI]	SEM (%)
L4–L5 level				
Right MF	0.993 [0.973, 0.998]	0.77	0.851 [0.513, 0.961]	3.92
Left MF	0.995 [0.982, 0.999]	0.65	0.877 [0.597, 0.968]	3.66
Right ES	0.983 [0.928, 0.996]	1.60	0.870 [0.556, 0.996]	4.44
Left ES	0.982 [0.934, 0.996]	1.39	0.894 [0.628, 0.973]	3.43
Right psoas	0.990 [0.961, 0.997]	0.29	0.962 [0.865, 0.990]	0.53
Left psoas	0.980 [0.903, 0.995]	0.32	0.944 [0.804, 0.984]	0.35
L5–S1 level				
Right MF	0.991 [0.962, 0.998]	0.85	0.962 [0.855, 0.990]	1.73
Left MF	0.995 [0.980, 0.999]	0.64	0.972 [0.895, 0.993]	1.6
Right ES	0.997 [0.914, 0.994]	0.60	0.944 [0.794, 0.986]	3.34
Left ES	0.974 [0.861, 0.994]	1.76	0.972 [0.866, 0.993]	2.09
Right psoas	0.944 [0.791, 0.986]	0.89	0.949 [0.819, 0.987]	0.82
Left psoas	0.963 [0.869, 0.991]	0.87	0.963 [0.865, 0.990]	0.42

### Correlation

**Fig. 3:**
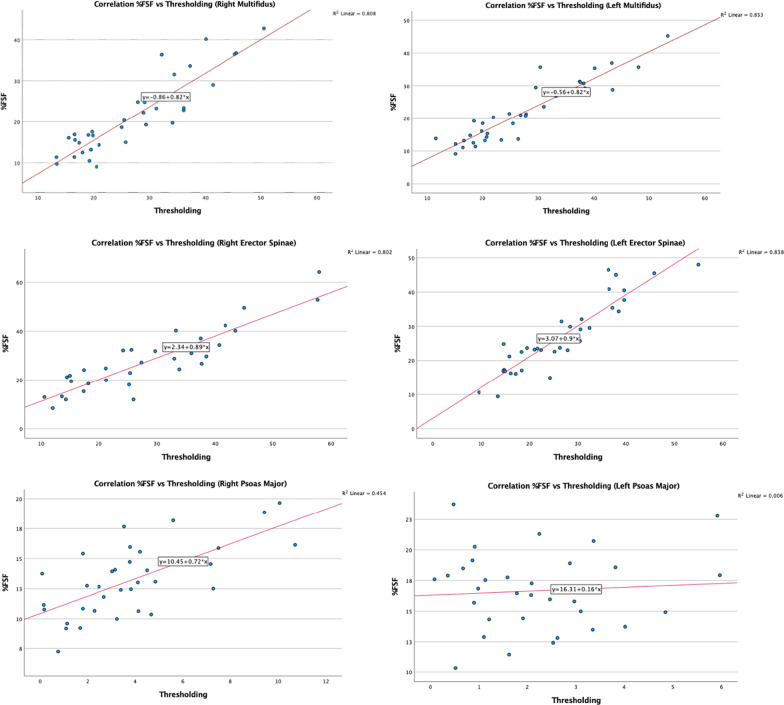
Correlation of multifidus, erector spinae and psoas major composition measurements obtained via the %FSF and thresholding methods at L4–L5

**Fig. 4:**
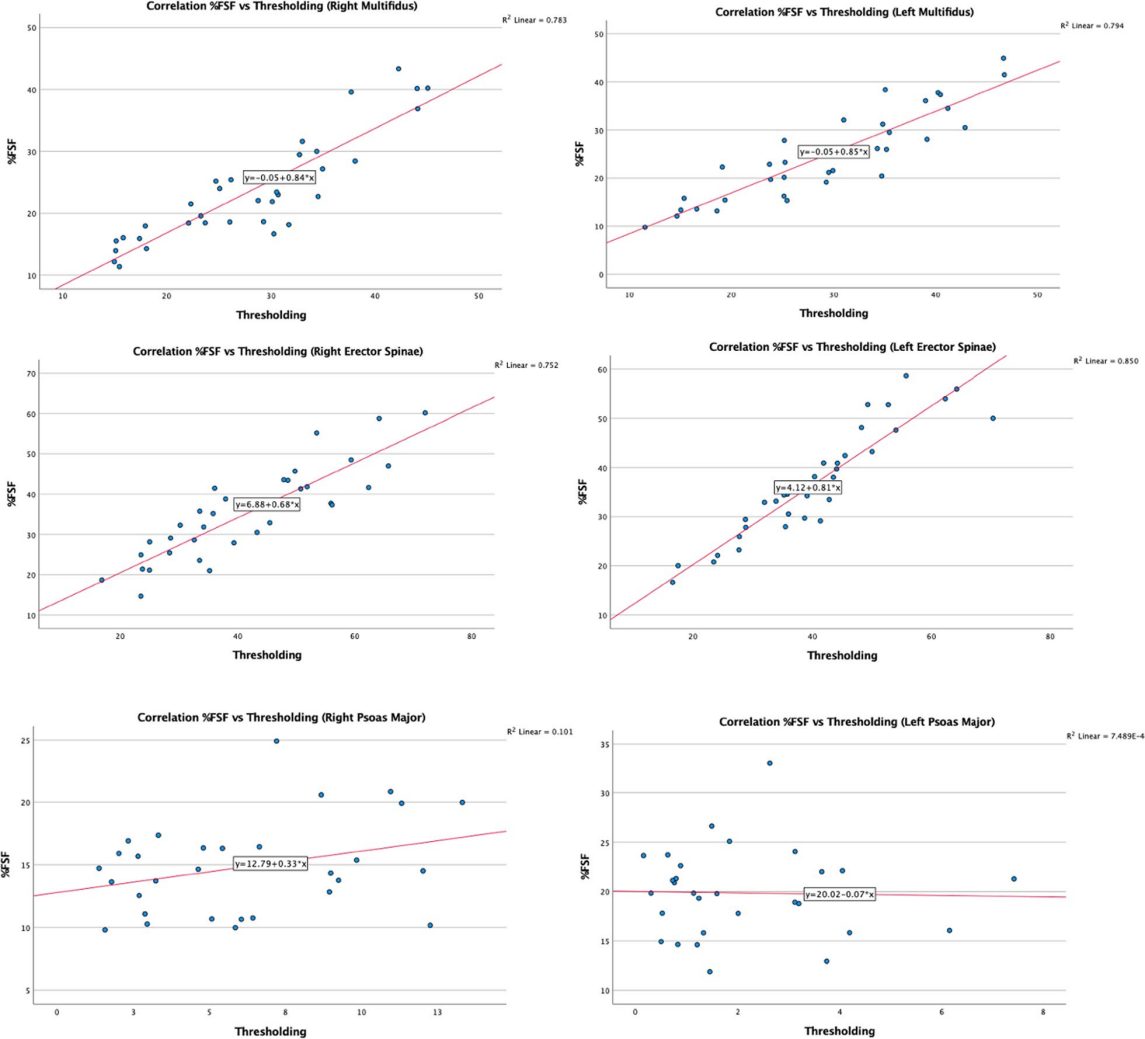
Correlation of multifidus, erector spinae, and psoas major composition measurements obtained via the %FSF and thresholding methods at L5–S1

The scatterplots demonstrating the correlation between muscle composition measurements obtained via the %FSF and thresholding methods are shown in Fig. 3 (e.g., L4–L5 level) and Fig. 4 (e.g., L5–S1 level). A strong positive correlation was found for both the MF and ES fat measurements bilaterally, and at both spinal levels. Pearson correlation coefficients (r) between both methods are presented in Table 3 and varied between 0.87 and 0.92. No correlation was found for the left PM fat measurements between both methods at the L4–L5 and L5–S1 level (r = 0.078 and − 0.027, respectively). The right PM fat measurements showed a moderate correlation at L4–L5 (r = 0.67) and a weak correlation at L5–S1 (r = 0.32) between methods.

**Table 3:** Pearson correlation coefficients between %FSF and thresholding measurements

Parameter	Mean ± SD			
	%FSF	Thresholding	r	95% CI
L4–L5 level				
Right MF	21.51 ± 9.24	27.38 ± 10.16	0.90	0.81–0.95
Left MF	21.87 ± 9.27	27.38 ± 10.45	0.92	0.85–0.96
Right ES	28.26 ± 12.29	29.07 ± 12.34	0.90	0.80–0.95
Left ES	27.03 ± 10.40	26.55 ± 10.55	0.92	0.84–96
Right psoas	13.25 ± 2.93	3.88 ± 2.73	0.67	0.44–0.82
Left psoas	16.67 ± 3.05	2.27 ± 1.50	0.078	− 0.26 to 0.40
L5–S1 level				
Right MF	23.67 ± 8.47	28.08 ± 8.88	0.89	0.78–0.94
Left MF	24.79 ± 9.12	29.25 ± 9.58	0.89	0.79–0.94
Right ES	35.44 ± 11.09	41.91 ± 14.11	0.87	0.75–0.93
Left ES	36.59 ± 10.93	40.25 ± 12.50	0.92	0.85–0.96
Right psoas	14.82 ± 3.77	6.14 ± 3.65	0.32	− 0.05 to 0.61
Left psoas	19.88 ± 4.50	2.15 ± 2.17	− 0.027	− 0.39 to 0.34

*CI* confidence interval

### Bland–Altman plots

**Fig. 5:**
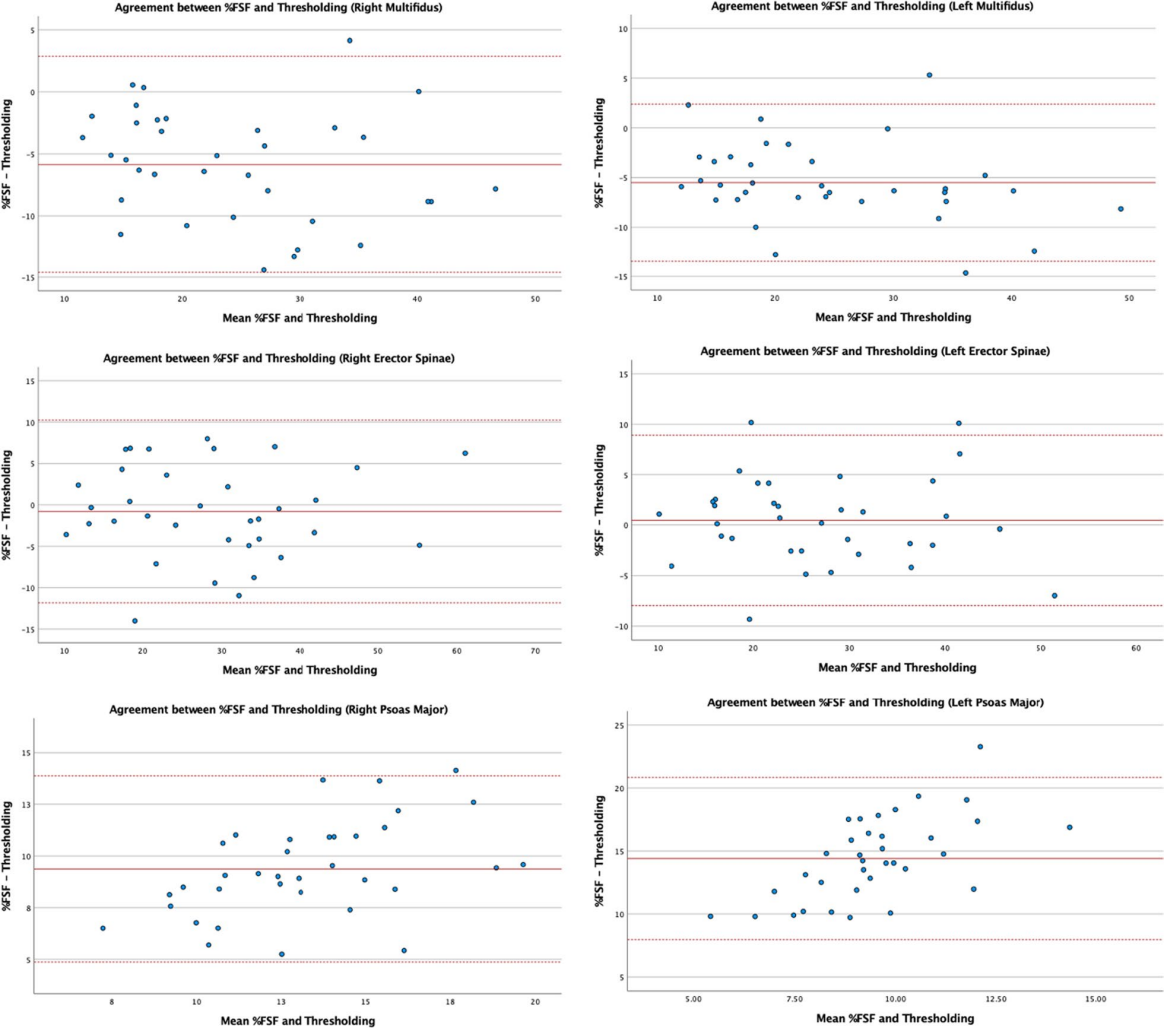
Bland-Altmann 95% limits of agreement plots for multifidus, erector spinae, and psoas major composition measurements obtained via the %FSF and thresholding method at L4–L5

**Fig. 6:**
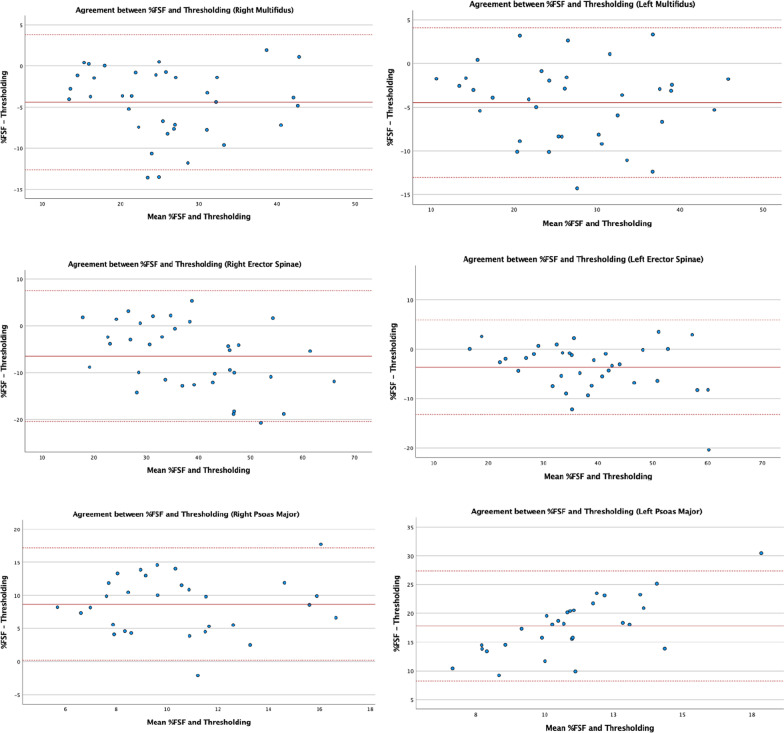
Bland-Altmann 95% limits of agreement plots for multifidus, erector spinae, and psoas major composition measurements obtained via the %FSF and thresholding method at L5–S1

The Bland–Altman 95% limits of agreement plots for bilateral muscle composition measurements at L4–L5 and L5–S1 are shown in Figs. 5 and 6, respectively. The Y-axis represents the mean difference between fat percentage measurements from the %FSF and thresholding methods and is plotted against the X-axis which represents the average of fat percentage measurements obtained from both methods. The Bland–Altman plots estimate possible bias based on the mean difference between two measurements and includes the limits of agreement (represented by dotted lines) that should encompass 95% of the data points [21]. Overall, Bland–Altman plots for the MF and ES show a mean difference close to zero and the data points are spread evenly above and below while staying dispersed within the 95% limits of agreement. All plots for the PM show data points evenly spread above and below the mean difference, however the mean difference is much higher than zero indicating greater measurement differences between methods as compared to the MF and ES measurements.

## Discussion

Previous research has demonstrated a link between fatty infiltration and LBP such that the presence of fat likely alters muscle structure and interferes with its function, thereby resulting in muscle dysfunction [8, 9, 22]. The purpose of this study was to assess the agreement between MF, ES, and PM muscle composition measurements obtained from fat–water and T2-weigthed axial images using %FSF and thresholding method, respectively. The muscle composition measurements were performed using two open-source image analysis software, Horos and ImageJ. Although both methods used were shown to be highly reliable [11, 12], their agreement had yet to be established. The correlation and agreement analysis of the paraspinal muscles and spinal levels investigated in this study suggest that both measurement methods yield comparable results, when applied in a clinically relevant population with chronic LBP.

The variability in paraspinal measurement methodologies and segmentation protocols employed across studies contributes to inconsistencies in the overall findings related to spinal muscle morphology and pathological conditions [11, 14–16]. This makes it difficult to compare results between studies as well as establish relationships between spinal pathologies and paraspinal muscle morphology. While paraspinal muscle composition measurements using %FSF and thresholding methods are highly reliable [12, 16], some differences still exist between them. Manual thresholding techniques demonstrate greater dependence on the rater as the rater must first choose an appropriate pixel value for lean muscle through the software’s histogram function before outlining the ROI [12, 23]. In contrast, the %FSF method allows for easier detection of fat infiltration due to higher signal contrast without the need of applying a threshold value [24]. Percent FSF measurements acquired via fat- and water-only images are most accurate for quantifying fatty infiltration as this MR sequence allows for a clearer visual of anatomical features and muscle fat infiltration than T2-weighted images [11, 15, 24]. Although the %FSF method is the contemporary standard for assessing muscle quality, T1- and T2-weighted images are widely used compared to fat- and water-only images, as they are more clinically accessible [11, 12, 15]. Therefore, assessing the agreement between the paraspinal muscle composition measurements acquired via different MR image sequences and related measurement methods was necessary to determine whether both methods were equally effective at assessing fatty infiltration. As a result, this will facilitate comparison of data between studies using either measurement methods of segmentation and help towards the standardization of methodologies.

Paraspinal muscle segmentation protocols are also a source of variation between studies. [11, 14, 15]. Most differ in whether they *include* or *exclude* the fat that may be present between the muscle border and its fascial attachments (e.g., epimuscular fat) [11, 14, 25]. Considering that epimuscular fat may potentially affect the integrity of a muscle, it should also be included with the intramuscular fat within the ROI to provide an accurate assessment of a muscle’s overall quality [11, 14, 25]. To achieve equal comparisons between studies, measurement methods and segmentation protocols should be clearly outlined and consistent.

In a study by Cooley et al. [26], measurements of MF muscle size and composition utilizing T1- and T2-weighted sequences showed excellent intra-rater reliability, demonstrating that both sequences are equally consistent for obtaining muscle measurements when conducted by an experienced examiner. In addition, no significant bias was detected when assessing the level of agreement between the two sequences [26]. As such, both T1- and T2- weighted could be used interchangeably for assessing paraspinal muscle morphology. However, in cases of severe muscle atrophy, edema or less muscle present, T1-weighted sequences may exhibit a higher signal intensity, resulting in higher fat measurements than T2-weighted sequences [26]. By selecting high-quality images for future studies, this bias could be mitigated [26].

### Correlation analysis and reliability

Pearson’s correlation coefficient was calculated, and scatter plots were conducted to examine the relationship between fat measurements obtained from both imaging methods. As shown in Figs. 1 and 2, the data points for the MF and ES muscles demonstrate less scatter and are closer to the line of regression, indicating a strong linear correlation between muscle composition measurements acquired via both methods. In the scatterplots for the right PM muscle measurements at both spinal levels, the data points show scatter with a positive linear correlation, whereas the left-sided measurements display significant scatter with no correlation. It is important to note the side-to-side differences in measurements which may be due to higher or lower signal intensity in areas where the amount of fat or water signal is ambiguous or a result of magnetic susceptibility [27].

We are not aware of any previous studies that compare measurements obtained using the %FSF method and the thresholding method. Intra-rater ICC values for the fat measurements obtained using the %FSF method obtained were excellent and comparable to other studies based on fat- and water-only images. For example, a study by Abbott et al. [28] demonstrated excellent intra-rater reliability for muscle fat infiltration measurements of the cervical multifidus muscle with ICC value equal to 0.98 and 95% confidence interval ranging between 0.97 and 0.98. Additionally, the ICC values for the lumbar multifidus fat measurements in a study by Rummens et al. [29] ranged between 0.985 and 0.998, indicating excellent intra-rater reliability. The consistency of ICC results across studies supports the clinical validity of the %FSF method for evaluating muscle quality. However, while the correlation coefficient is useful for assessing the strength of a relationship between two measurements, it does not assess the difference between measurements to determine whether both variables show agreement [20, 30].

### Bland–Altman analysis

The Bland–Altman plots were used to examine the degree of agreement between the two methods and detect possible systematic bias in the data. Overall, plots for the MF and ES measurements showed no systematic bias, as all data points were evenly distributed above and below the mean difference and 95% were located within limits of agreement. However, the Bland-Altmann plot for bilateral PM at both levels suggests systematic bias as the mean difference is further from zero with greater differences in fat measurements. In general, the fat percentage measurements of the PM muscle were greater using the %FSF as compared to the thresholding technique.

In addition to the MF and ES plots illustrating data points within the 95% limits, if the width of the limits of agreement between the two measurement methods were relatively small and within an acceptable range suggesting that both methods could be used interchangeably when examining the morphology of the MF and ES [31, 32]. However, PM fat values obtained using the %FSF and thresholding method do not appear to agree as there are large differences between the measurements. While current literature lacks a clear definition of what constitutes a small width, our limits of agreements for MF and ES were comparable to a previous measurement study assessing the agreement between T1- and T2-weighted paraspinal muscle composition measurements [26].

The fat measurements obtained in our study for the PM muscles were lower compared to the MF and ES, a finding that may be attributed to increased activation of the PM to compensate for reduced activity in the MF and muscle atrophy [33, 34]. In fact, it is rare to see visible fat in the PM muscle on T2-weighted images. Similarly, Arbanas et al. [33] evaluated the PM using T2-weighted sequences and found low levels of fatty infiltration in patients with LBP, which was comparable to controls. Therefore, the PM likely remains active whether LBP is present or not and also plays a stabilizing role [33]. A study by Fortin et al. [35] reported low fatty infiltration in the PM compared to the MF at the same spinal level in patients with lumbar spinal stenosis. This could be attributed to denervation of the MF which leads to disuse of the entire muscle, as it is only innervated by a single nerve root, and consequently increased atrophy and fatty infiltration over time [11, 35, 36]. This finding has not been observed in the PM, which is innervated by multiple nerve roots [35]. Nevertheless, the lower level of visible intramuscular fat present in the PM muscle may partly explain the discrepancy between the two measurement methods.

### Limitations

This study has some limitations. First, the MF, ES, and PM muscles were only assessed at the two lower spinal levels. Future studies should investigate paraspinal muscle composition at additional spinal levels as well as analyse other muscles that may be related to LBP, such as the quadratus lumborum muscle [6]. Moreover, only individuals with back pain were included in our study, thus our findings cannot be generalized to healthy asymptomatic individuals.

## Conclusion

In conclusion, the correlation and Bland–Altman agreement analysis of the paraspinal muscles and spinal levels investigated in this study suggest that both methods yield comparable measurements for the MF and ES, when applied to a clinically relevant population. Clinically, our findings suggest that there are no important concerns with using T2-weighted or IDEAL fat–water sequences interchangeably to investigate MF or ES paraspinal muscle composition, when measurements are obtained by experienced examiners. However, we found inconsistencies and disagreement for the assessment of PM composition between each method, suggesting that muscle with lower fat content may lead to wider disagreements between T2-weighted and IDEAL fat–water composition measurements. While our findings are promising, further research is needed to confirm and expand our results to other paraspinal muscles, spinal levels, and populations, including healthy asymptomatic individuals. Reducing measurement variability and using standardized accurate paraspinal muscle composition measurement methods will facilitate comparison among studies.

## Data Availability

The datasets generated and/or analysed during the current study are not publicly available due to the nature of this study, but are available from the corresponding author on reasonable request.
